# Accuracy of Video-Based Hand Tracking for People With Upper-Body Disabilities

**DOI:** 10.1109/TNSRE.2024.3398610

**Published:** 2024-05-15

**Authors:** Alexandra A. Portnova-Fahreeva, Momona Yamagami, Adrià Robert-Gonzalez, Jennifer Mankoff, Heather Feldner, Katherine M. Steele

**Affiliations:** Department of Computer Science and Engineering, University of Washington, Seattle, WA 98105 USA; Department of Computer Science and Engineering, University of Washington, Seattle, WA 98105 USA.; Department of Electrical and Computer Engineering, Rice University, Houston, TX 77005 USA; Department of Rehabilitation Medicine, University of Washington, Seattle, WA 98105 USA; Department of Computer Science and Engineering, University of Washington, Seattle, WA 98105 USA; Department of Rehabilitation Medicine, University of Washington, Seattle, WA 98105 USA; Department of Mechanical Engineering, University of Washington, Seattle, WA 98105 USA

**Keywords:** Dimensionality reduction, hand tracking, principal component analysis, synergies, upper-body disabilities, hand therapy, rehabilitation

## Abstract

Utilization of hand-tracking cameras, such as Leap, for hand rehabilitation and functional assessments is an innovative approach to providing affordable alternatives for people with disabilities. However, prior to deploying these commercially-available tools, a thorough evaluation of their performance for disabled populations is necessary. In this study, we provide an in-depth analysis of the accuracy of Leap’s hand-tracking feature for both individuals with and without upper-body disabilities for common dynamic tasks used in rehabilitation. Leap is compared against motion capture with conventional techniques such as signal correlations, mean absolute errors, and digit segment length estimation. We also propose the use of dimensionality reduction techniques, such as Principal Component Analysis (PCA), to capture the complex, high-dimensional signal spaces of the hand. We found that Leap’s hand-tracking performance did not differ between individuals with and without disabilities, yielding average signal correlations between 0.7–0.9. Both low and high mean absolute errors (between 10–80mm) were observed across participants. Overall, Leap did well with general hand posture tracking, with the largest errors associated with the tracking of the index finger. Leap’s hand model was found to be most inaccurate in the proximal digit segment, underestimating digit lengths with errors as high as 18mm. Using PCA to quantify differences between the high-dimensional spaces of Leap and motion capture showed that high correlations between latent space projections were associated with high accuracy in the original signal space. These results point to the potential of low-dimensional representations of complex hand movements to support hand rehabilitation and assessment.

## I. Introduction

There is a large number of conditions that may require an individual to participate in upper-extremity rehabilitation that includes assessments of hand function: from cervical spinal cord injuries (166,000 in US) and stroke (795,000/year in US) to Parkinson’s disease (one million individuals in US), various forms of arthritis (58.5 million individuals in US), and carpal tunnel syndrome (about 10mil in US) [1], [2], [3], [4], [5]. As with any form of medical treatment, constant visits to the office, as well as laborious procedures, can lead to patient/provider burnout. In addition, assessing function in a biological structure as complex as the human hand is a difficult task for providers who must consider all 27 degrees-of-freedom (DOFs) in the hand alone. Recent advances in hand-tracking technologies offer the potential to make more cost-effective and engaging options for routine hand function assessments and interventions as well as provide clinicians with tools for more objective data on outcomes following rehabilitation.

There are numerous hand-tracking technologies available: from expensive sensorized gloves (*e.g*., CyberGlove (*Cyber Glove Systems, San Jose, CA, USA*)) to cheaper camera-based solutions. In this paper, we will be evaluating Leap (*Ultraleap, Bristol, England*), a small device (145mm x 18.6mm x 11.1mm) with two infrared cameras that performs image detection to track hands. In the recent decade, many research groups have proposed the utilization of the device’s hand-tracking feature for the purpose of hand rehabilitation, especially for stroke and Parkinson’s disease [6], [7], [8], [9], [10], [11], [12], [13], [14], [15], [16], [17]. These groups have used Leap to explore incorporating virtual gaming into hand rehabilitation to practice grabbing, reaching, pointing, lifting, and other tasks. The main advantages of Leap are its cost-effectiveness ($100–250/piece) and ease of integration with available programming systems that make it an appealing piece of technology for the use in research environments. In addition, its pre-packaged hand-tracking module and the use of built-in infrared cameras and infrared LEDs to illuminate the hand for improved tracking make Leap a more suitable choice over integrating RGB-D cameras with one of many existing object-tracking technologies.

However, the accuracy of assessment and rehabilitation intervention activities with Leap has not been formally established with individuals with disabilities, a critical next step if the device is to be recommended to supplement or enhance traditional hand rehabilitation. Past work has been performed either with a static plastic hand [18], [19] or nondisabled individuals [20], [21], [22], [23], [24], [25]. Further, the early assessment studies report accuracy only across a small subsection of the high-dimensional data available for the human hand, or display full signal data, but only for one participant. There, accuracy is reported in terms of static poses, possible ranges of motions of each finger, or hand segment estimation at a single time instance [20], [22], [24]. We believe that such approaches, while easy to implement and conventional in their nature, are inefficient for proper accuracy evaluation and insufficient for what they are being used for – insight and evaluation of one’s hand function. This is because they require individual evaluation of every DOF instead of providing an overall picture of hand-tracking performance that is indicative of the higher-dimensional signals. We suggest that other techniques must be developed and evaluated to provide more insightful evaluations of high-dimensional signal comparison to complement and enhance upper-extremity rehabilitation practices.

As a result, in this paper, our objectives were to 1) assess the accuracy of Leap’s hand-tracking feature for individuals with and without upper-body disabilities and 2) propose and evaluate a new technique to assess high-dimensional hand-tracking data via dimensionality reduction.

## II. Methods

### A. Participants

Participant recruitment and participation were approved by the University of Washington’s Institutional Review Board (STUDY00016152), approved in November 2022, and informed written consent was obtained from each study participant. We recruited individuals with and without upper-body disabilities. The inclusion criteria included sufficient hand dexterity to perform fingertip touches and finger spreading. Disabled participants reported conditions such as motor neuron disease, muscular dystrophy, essential tremor, spinal cord injury, arthritis, stroke, multiple sclerosis, and Charcot-Marie-Tooth disease.

**Fig. 1.**
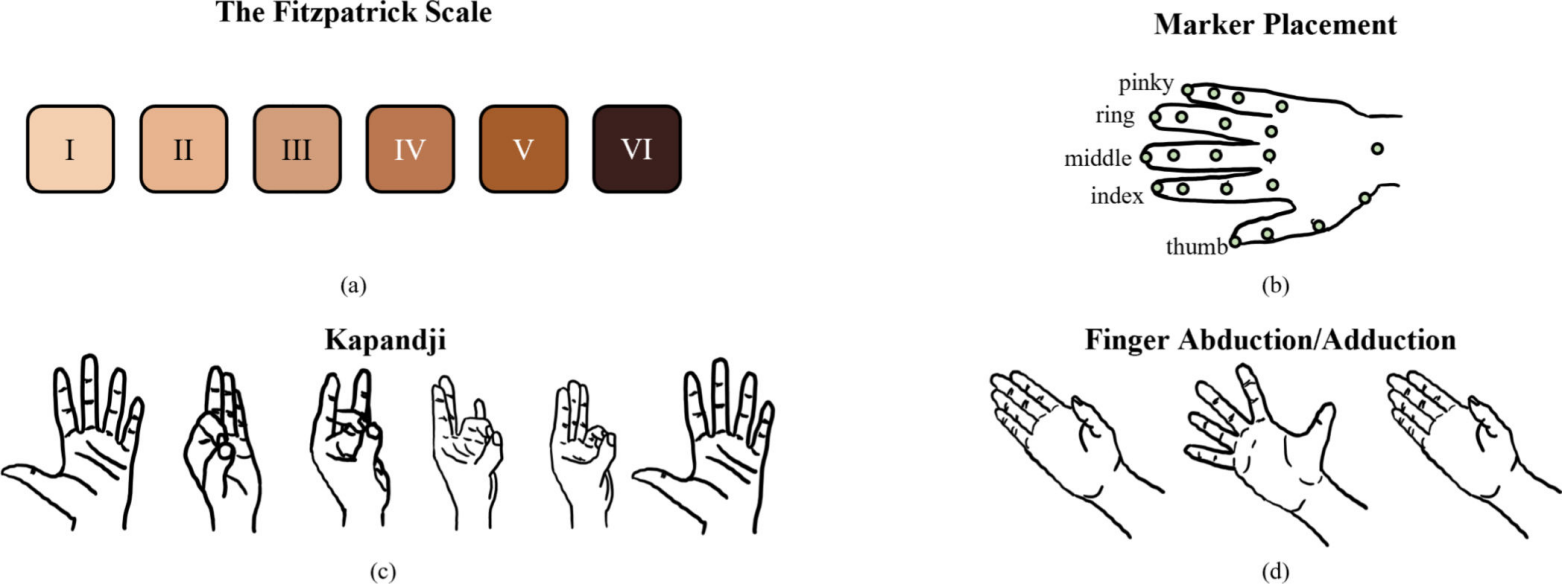
**(a)** The Fitzpatrick scale for skin tone self-assessment. **(b)** Placement of the 21 markers on the hand. **(c)** Kapandji task. **(d)** Finger abduction/adduction task.

A total of 10 disabled (6 females, 3 males, 1 non-binary, 52.5±20.4 y/o) and 7 nondisabled (5 females, 2 males, 30.4±11.6 y/o) participants were recruited for the study. The disabled group was asked to fill out a Quick Disabilities of Arm, Shoulder & Hand (QuickDASH) assessment, which is designed to measure physical function and symptoms of the upper-limb module [26]. The questions were slightly modified to alleviate ableism assumptions that the person’s disability was a *problem*. For example, a question asking “…to what extent has your arm, shoulder, or hand *problem* interfered with your normal social activities…?” was replaced by “…to what extend your arm, shoulder, or hand *affected by your disability and/or chronic condition* interfered with your normal social activities…?” The average QuickDASH score was 43.6 ± 17.6. Participants in both groups were also asked to identify their skin tone according to the Fitzpatrick Classification scale (Fig. 1a). This information was recorded in order to assess the dependence of the accuracy of camera-based hand-tracking solutions, such as Leap, on the user’s skin tone. Participants self-identified between levels I and IV, with the following breakdown for each level: I – four participants, II – three participants, III – five participants, IV – five participants. In the past, it has been reported that the accuracy of worn sensors is lower for individuals with darker skin tones [27], [28], [29].

### B. Experimental Setup & Protocol

For accuracy assessment, we evaluated Ultraleap’s high-performance Stereo IR 170 Camera Module (formerly known as Rigel). When compared to the more-general-use Leap Motion Controller, the Stereo IR 170 incorporates a wider field of view (170°×170°) and a longer tracking range (between 10cm to 75cm preferred, up to 1m maximum). We will continue to refer to this module as Leap throughout the paper. Leap performed hand tracking using the Gemini software (*Ultraleap, Bristol, England*), version 3.1.0. When evaluated with an industrial robot, Leap yielded average accuracy of 1.2mm when tracking a single point [30].

Accuracy of Leap was compared against an Optitrack motion capture system (*NaturalPoint, Inc., Corvallis, OR, USA*), with measurement errors of under 0.1mm. The setup consisted of 14 Prime^x^ 13W cameras, mounted on a 6 × 6ft canopy and directed towards a small 3×3ft area by the table, where the participant was seated in front of a computer display (Supplementary Fig. 1). Leap was secured to the table in the face-up configuration. All data collection and synchronization were implemented in Unity (*Unity Technologies, San Francisco, CA, USA*).

A total of 21 4mm reflective markers were placed on each participant’s hands on the joint centers and the fingertips (Fig. 1b). For index, middle, ring, and pinky fingers, markers were placed on the metacarpophalangeal (MCP), proximal interphalangeal (PIP), and distal interphalangeal (DIP) joints as well as the most distal point of each digit’s dorsal side. For the thumb, markers were placed on the most distal point on the dorsal side as well as the MCP, carpometacarpal (CMC), and interphalangeal (IP) joints. For the wrist joint, a marker was placed on the dorsal side of ulnocarpal joint. With both Optitrack and Leap, we recorded 3D positions of joints and fingertips. Unfortunately, due to an error in the code, the position of the thumb’s MCP was not recorded by the Leap, so we removed it from our analysis. Consequently, each system (Leap and Optitrack) yielded 60 signals (3D locations of 20 markers).

At the beginning of the experiment, we assessed the hand function of each participant using the Jebsen Taylor Hand Function (JTHF) Test [31] – a common assessment tool among physical and occupational therapists. We performed six out of seven subtests (card turning, picking up small objects, stacking checkers, simulated feeding, moving light objects, and moving heavy objects) for both hands. The writing subtest was avoided due to the time constraints on the experiment.

The participants then performed two tasks: a variation of Kapandji (touching the fingertip of each finger with their thumb once) (Fig. 1c) and finger abduction/adduction (Fig. 1d). These tasks are reflective of traditional tasks to assess range of motion of one’s hand and representative of common device interactions that are a combination of finger and thumb flexion/extensions and abduction/adduction. Each task was performed 5 times (trials) with each hand. The task was first demonstrated by the experiment coordinator and a corresponding picture appeared on the computer display in front of the participant. Participants were instructed to perform the tasks about 10–20cm above the Leap within a 40 × 40cm box, defined by blue tape, with the device in the middle. They were asked to keep their palm as parallel to the table as possible. P105 had sufficient hand dexterity to perform the desired tasks, but not enough muscle strength to keep their hands above the table, so one of the experiment coordinators provided support by holding their arms about 15cm above the desk such that participant’s hands were directly above the Leap and the Optitrack cameras were not occluded.

Data recording went as follows:

1. The participant held their hand flat on the table.
2. Following a “START” sound cue, the participant performed the task at a self-selected speed.
3. Upon completing the task, the participant placed their hand back on the table and data collection was stopped.
4. In case of faulty trials, steps 1–3 were repeated.

While for majority of the trials, we had 60 signals to compare, the CMC marker was misplaced for a few participants. During the Kapandji task, P123 had a misplaced CMC marker for both hands. During finger abduction/adduction, P105 had a misplaced CMC on the nondominant hand and P123 had a misplaced CMC on the dominant hand. As a result, during these instances, analyses were performed for the remaining 57 signals.

Static and dynamic parameters were measured for further detailed assessment. The static parameters were hand size (determined by measuring the distance between the wrist and the middle MCP markers when the palm of the hand was positioned flat on the table), time taken to perform the picking up of small objects subtest of the JTHF test, QuickDASH, and self-identified skin tone. The dynamic parameters were trial-dependent and included the average height of the wrist joint above the table throughout the task as well as the speed of task performance. For the Kapandji task, speed was determined by calculating the speed of the tip of the thumb as it was the main finger involved throughout the task. For finger abduction/adduction, speed was calculated from the index fingertip, as we assumed a uniform finger movement during the task.

### C. Data Processing

#### Optitrack:

The motion capture data were cleaned using Motive software (*NaturalPoint, Inc., Corvallis, OR, USA*) to eliminate dropped markers. Because each tracking system had unique detection parameters (*i.e*., Optitrack detected hands flat on the table prior to trial and Leap only detected them when they were in the device’s range), data were clipped so all comparisons were made with data tracked by both systems. Due to space limitation and the need to work around our participants’ accommodations such as various mobility aids, Optitrack yielded poor marker tracking during a few trials, which were consequently dropped from analysis.

#### Leap:

Although data were sampled at 50Hz from Leap and Optitrack, due to the Leap’s variability in sampling frequency, the final number of samples recorded at the end of each trial for Leap and Optitrack often did not match. The nonuniformly sampled Leap data were resampled to match that of the uniformly sampled Optitrack data. To align the Leap and Optitrack systems, the Leap data were offset in the x, y, and z directions so that the position of the MCP joint of the index finger determined by Leap during the first analysis instance matched that of the index’s MCP joint determined by Optitrack. This was done to align the two coordinate systems and account for any potential offsets that might have happened during system calibration. Relative orientations were aligned during the motion capture calibration step by placing the Optitrack calibration square so that the Optitrack coordinate axes aligned with those of Leap.

#### Hand Dominance:

Hand dominance reported by unimpaired individuals was used during analysis. For individuals without a disability, the dominant hand tends to yield faster performance on the JTHF test [31]. However, for individuals with a disability, there was significant variability in how participants reported hand dominance. For example, some reported their dominant hand as the hand that was least affected by their current condition, regardless of handedness prior to disability onset, while some reported their hand dominance prior to the disability. Thus, we decided to determine hand dominance based on the results of the “picking up small objects” subtest of the JTHF test – the hand that performed the task faster was assigned as the dominant hand for analyses. We picked this subtest for its most appropriate representation of hand dexterity.

### D. Accuracy Assessment

#### Conventional Techniques:

Two of the most common techniques to compare two signals are correlation coefficients and mean absolute error (MAE). We started the accuracy assessment of hand tracking by determining the correlation, $corr_{i}$, of each $i^{th}$ signal between Leap and Optitrack, where each signal represents a joint/tip position either along the x, y, or z axis. For each participant and task, we calculated the average of all correlations, $corr_{signal}$, where n is the total number of signals in the original data (1).


(1) $$corr_{signal}=\frac{\sum_{i=1}^{n}corr_{i}}{n}$$

We then calculated MAE for each signal pair, $signal_{LEAP,i}$ and $signal_{Optitrack,i}$ at every time instance t (2).


(2) $$MAE_{i}=\frac{\sum_{1}^{t_{last}}\left|signal_{LEAP,i}(t)-signal_{Optitrack,i}(t)\right|}{t_{last}}$$

As with correlation, we calculated the average MAE across all signals, $MAE_{avg}$ (3).


(3) $$MAE_{avg}=\frac{\sum_{i=1}^{n}MAE_{i}}{n}$$

These conventional accuracy assessment methods are often not optimal for signal comparison. Ideally, when comparing Optitrack and Leap for accuracy, we want to see two signals that exhibit high correlation and low MAE. However, there are several examples when these two methods may lead to contradicting results. For example, two signals that match in temporal pattern, but have a large offset in the amplitude will yield high correlation but also high MAE (Supplementary Fig. 2a). In other instances, two signals might yield low MAE (desirable), while resulting in low correlation (Supplementary Fig. 2b). Or there might be a temporal offset, as one signal lags behind the other –yielding low correlation and, potentially, low MAE, yet there would be no indication of the temporal lag of the signal in these outcomes (Supplementary Fig. 2c).

Moreover, these conventional techniques are not efficient for comparison of high-dimensional data, requiring multiple steps of average calculations. Such mathematical manipulations result in outcomes that may not be indicative of finer details that are important for assessment or monitoring of hand function.

In addition to evaluating correlation coefficients for each hand landmark’s 3D position, we also evaluated the correlation of the Euclidian distances between digits for each task, $corr_{fingertip}$. Identifying functionally important measures from these tasks is one method to reduce the dimensionality of the high-dimensional data from the hand. For Kapandji, we calculated Euclidian distances between the tip of the thumb and each fingertip. For finger abduction/adduction, we evaluated the Euclidian distances between neighboring digit tips. In both cases, evaluating these distances reduced the available 60D signal space to 4D (Supplementary Fig. 3).

Lastly, we calculated the length of each finger segment (proximal, middle, distal)– a total of 12 segments per hand. The thumb was excluded from this analysis due to the missing data. The segment lengths were evaluated during the finger abduction/adduction exercise in the middle of each trial with a single frame selected for analysis. This assessment technique allowed us to analyze accuracy of the Leap-generated hand model and the percent match of the recorded segments compared to the hand segments measured with Optitrack.

#### Dimensionality Reduction:

While calculating measures like Euclidian distances between fingertips is one method to reduce the dimensionality of available data for analysis, this choice draws from only a limited set of the original data and may not capture important features for a given individual and task. An alternative to manual selection of features is using dimensionality reduction (DR) techniques, which come in a variety of types – from complex neural networks to simple mathematical equations. In this study, we used one of the most common linear techniques – Principal Component Analysis (PCA) [32]. We chose PCA for its ease in implementation, low computational cost, and stability in output in comparison to other methods, such as autoencoders or t-Distributed Stochastic Neighbor Embedding (tSNE). Such latent space stability makes it possible to perform comparisons in the low-dimensional spaces. Due to its linear and orthogonal constrains, PCA yields latent spaces that are easy to interpret unlike its nonlinear counterparts. It is also one of the most common DR techniques applied in the biosignal context. PCA calculates the linear combinations of input signals that explain the most variance in the data. This method has been widely applied in the past to reduce the dimensionality of complex hand kinematics during object grasping, American Sign Language gesturing, and dynamic tasks [33], [34], [35], [36]. In most cases, PCA was able to explain over 80% of variance in the input data with just two latent dimensions, or principal components (PCs).

To evaluate the potential of PCA for visualization and analysis of these tasks, we first assessed the impact of common issues with video-based methods – noise, offsets, and delays. We used a dataset from our experimental data (P105 performing Kapandji with the dominant hand) and synthetically modified data to mimic these issues. To evaluate the impact of noise, we added Gaussian noise to all 60 signals ranging from small (signal-to-noise ratio of 50dB) to large (30dB). The encoding of the data injected with small noise appeared very similar to the original data encoding (Supplementary Fig. 4). Increasing the noise yielded a more distorted encoding. To evaluate an offset, we offset the amplitude of all 60 signals by 10mm. The offset in the dataset resulted in a linear transformation of the projection in the latent space (Supplementary Fig. 4). Finally, we included a novel dataset – the same participant performing the finger abduction/adduction task – to understand how data from different origins are organized on the latent space. In this case, the encoding of the novel data did not appear similar in shape to the original data encoding. This suggests that PCA can be used to determine differences between two datasets (e.g., Leap and Optitrack) since if data are the same in the high-dimensional space, their encodings will resemble each other in the lower-dimensional space.

To use PCA to evaluate the accuracy of Leap relative to Optitrack, each trial pair, $x_{Leap}$ and $x_{Optitrack}$ (both of which are m×n matrices, where m is the number of samples and n is the number of signals) were combined into a single high-dimensional data input, $x_{combined}$, resulting in an 2m×n matrix. The data were then normalized according to (4), where i is each individual signal.


(4) $$x_{norm,i}=\frac{x_{combined,i}-\min\left(x_{combined,i}\right)}{\max\left(x_{combined,i}\right)-\min\left(x_{combined,i}\right)}$$

The normalized data, $x_{norm}$, were then passed through PCA and the two PCs that explained the greatest variance were extracted and plotted in 2D space. To visually differentiate between Leap and Optitrack latent projections, encodings associated with Leap were plotted in red while those generated from Optitrack samples were plotted in blue Once in 2D, the Leap projection was offset by $x_{offset}$, $y_{offset}$, such that the mean of the data along the first latent dimension (PC1) and the mean of the data along the second latent dimension (PC2) matched that of Optitrack’s encoding (5)–(6). This was done to visually assess the difference in latent space projections between Leap and Optitrack.


(5) $$x_{offset}=\mu_{Optitrack,PC1}-\mu_{Leap,PC1}$$

(6) $$y_{offset}=\mu_{Optitrack,PC2}-\mu_{Leap,PC2}$$

### E. Statistical Analysis

For statistical analyses, we used custom scripts using MATLAB’s (*MathWorks, Natick, MA, USA*) Statistics Toolbox. A Shapiro-Wilk test [37], [38] was used to evaluate normality of mean correlation, MAE, Euclidian distances, and error between finger segments. This analysis indicated the distributions were normal for the mean correlation and MAE values and not normal for the Euclidian distances and errors between finger segments. Subsequently, for mean correlation and MAE, we used Holm-Sidak test [39], [40] to account for multiple comparisons. For not-normally distributed data, such as Euclidian distances and absolute errors between estimated finger segments, we utilized Dunn’s test [41], [42], a procedure used for multiple non-parametric comparisons. We used stepwise regression [43] to evaluate the effects of static and dynamic parameters on signal correlations.

## III. Results

### A. Accuracy Assessment Indicates no Significant Difference Between Disabled and Nondisabled Hand Tracking With Leap

**Fig. 2.**
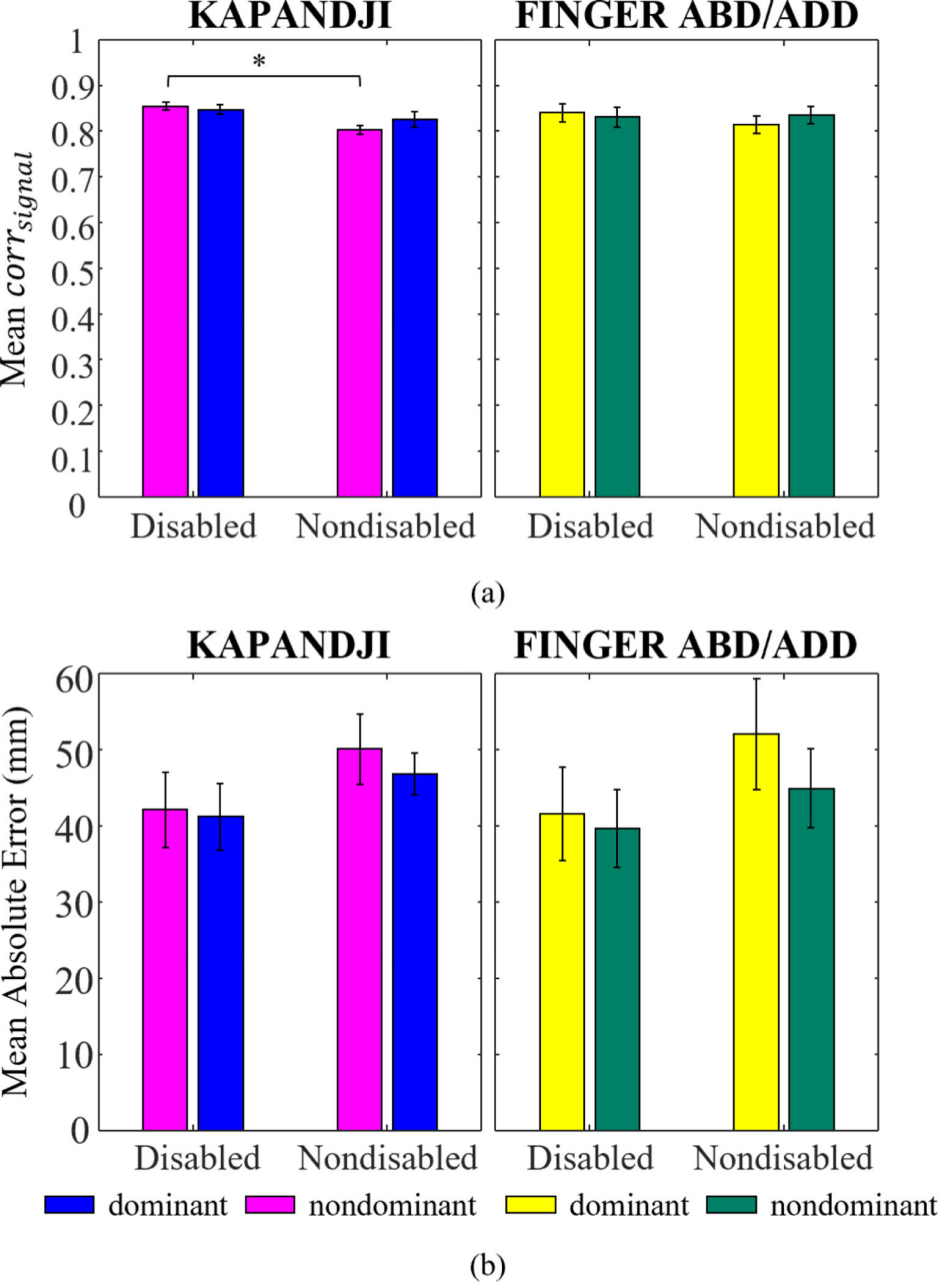
**(a)** Correlation, $corr_{signal}$ and **(b)** mean absolute error (MAE) of digit landmark trajectories between Leap and Optitrack averaged across all signals for each participant in the disabled (N=10) and nondisabled (N=7) groups during Kapandji and finger abduction/adduction tasks. Averages and standard errors across participants are plotted. * indicates significant difference in the mean correlation for nondominant hand between disabled and nondisabled participants (*p* = 0.003, Holm-Sidak test).

Using the high-dimensional set of signals from hand landmarks, we found high correlations, $corr_{signal}$, (between 0.7–0.9) comparing Leap and Optitrack during both the Kapandji and finger abduction/adduction tasks (Fig. 2a). Leap’s performance did not differ between dominant and nondominant hands (p > 0.05). The disabled group yielded significantly higher $corr_{signal}$ between Leap and Optitrack for nondominant hands during the Kapandji tasks (*p* = 0.003). In other cases, there were no significant differences in mean $corr_{signal}$ across both groups. MAEs were similar between dominant and nondominant hands and for both groups (p > 0.05, Fig. 2b). MAE results showed a greater variability with a range of 10–80mm error between the Leap and the Optitrack signals. There were no significant differences between the hand dominance or disability status variables (p>0.05). Following these results, both participants from disabled and nondisabled groups were combined in the following analysis as no statistical differences were observed between them.

We found limited to no dependency of the $corr_{signal}$ between Leap and Optitrack on static (*i.e*., skin tone, hand measurements, and the picking up small objects subtests of JTHF Test) or dynamic (*i.e*., hand height above the Leap and the speed of task performance) parameters. During the Kapandji task, task speed was negatively associated with $corr_{signal}$ between Leap and Optitrack for the disabled group, such that accuracy of the Leap decreased with the increase in the speed with which the participant performed the task (*p* < 0.01, $R^{2}=0.62$). During the same task, $corr_{signal}$ for the dominant hand for the nondisabled group depended on the hand position above Leap (*p* = 0.01, $R^{2}=0.74$), such that performing the task further away from Leap resulted in more accurate tracking of the hand. For the finger abduction/adduction task, the performance of the Leap’s hand-tracking feature was not associated with any of the static or dynamic parameters. QuickDASH scores were not associated with average correlation values for either of the tasks.

**Fig. 3.**
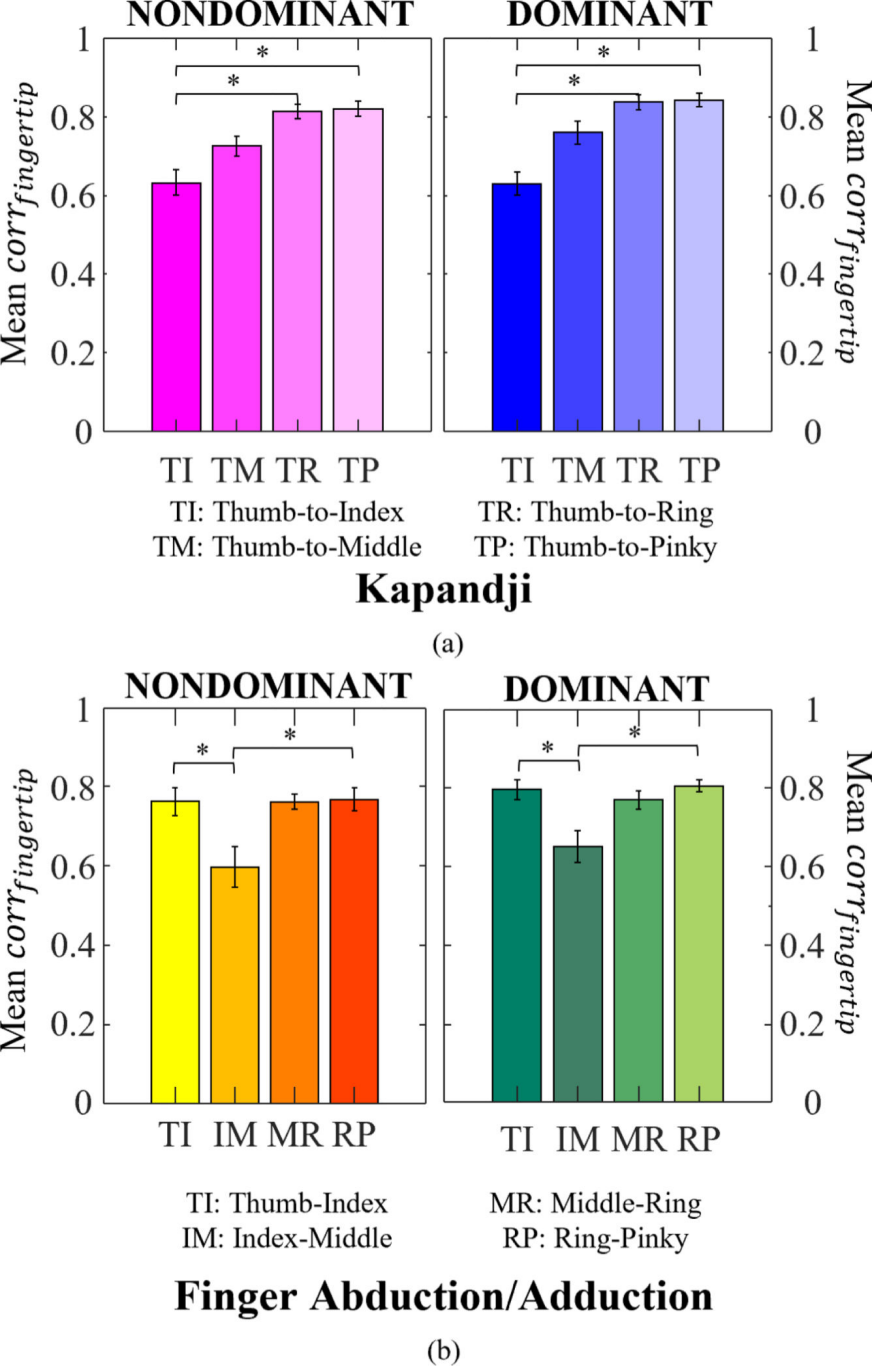
Correlation $\left(corr_{fingertip}\right)$ between Euclidian distances calculated between (a) thumb tip and other fingertips for the Kapandji task and (b) neighboring fingertips during the finger abduction/adduction task. Average correlation was calculated for each participant across all trials, and then averaged across participants. The graph presents average and standard errors. * indicate statistical significance with p < 0.01, Dunn’s test for multiple comparisons.

Evaluating Euclidian distances between digits during the Kapandji demonstrated overall acceptable performance of Leap’s hand-tracking of the general fingertip positions in relationship to each other (Fig. 3a). Leap exhibited the lowest performance tracking the index finger (average correlation of 0.63 for both hands). A better performance could be seen for the middle finger (mean $corr_{fingertip}$ of 0.76, *p* > 0.05, for dominant and 0.73, *p* > 0.05, for nondominant hands). The ring and pinky fingers had the highest correlations, with a mean $corr_{fingertip}$ of 0.81 for dominant and 0.84 for nondominant hands. The differences between both index and ring and index and pinky pairs were statistically significant for dominant and nondominant hands alike (*p* < 0.01).

During the abduction/adduction task, the Euclidian distances between the neighboring fingertips had high $corr_{fingertip}$ (>0.76) between Leap and Optitrack for all digit pairs except for between the index-and-middle (IM) which had an average $corr_{fingertip}$ of 0.65 for dominant and 0.60 for nondominant hands (Fig. 3b). The differences between the thumb-to-index (TI) and IM pairs were statistically significant for both hands (*p* = 0.008 for dominant and *p* = 0.004 for nondominant hands). Similarly, the differences between the IM and ring-pinky (RP) pairs were statistically significant (*p* = 0.001 for dominant and *p* = 0.003 for nondominant hands).

**Fig. 4.**
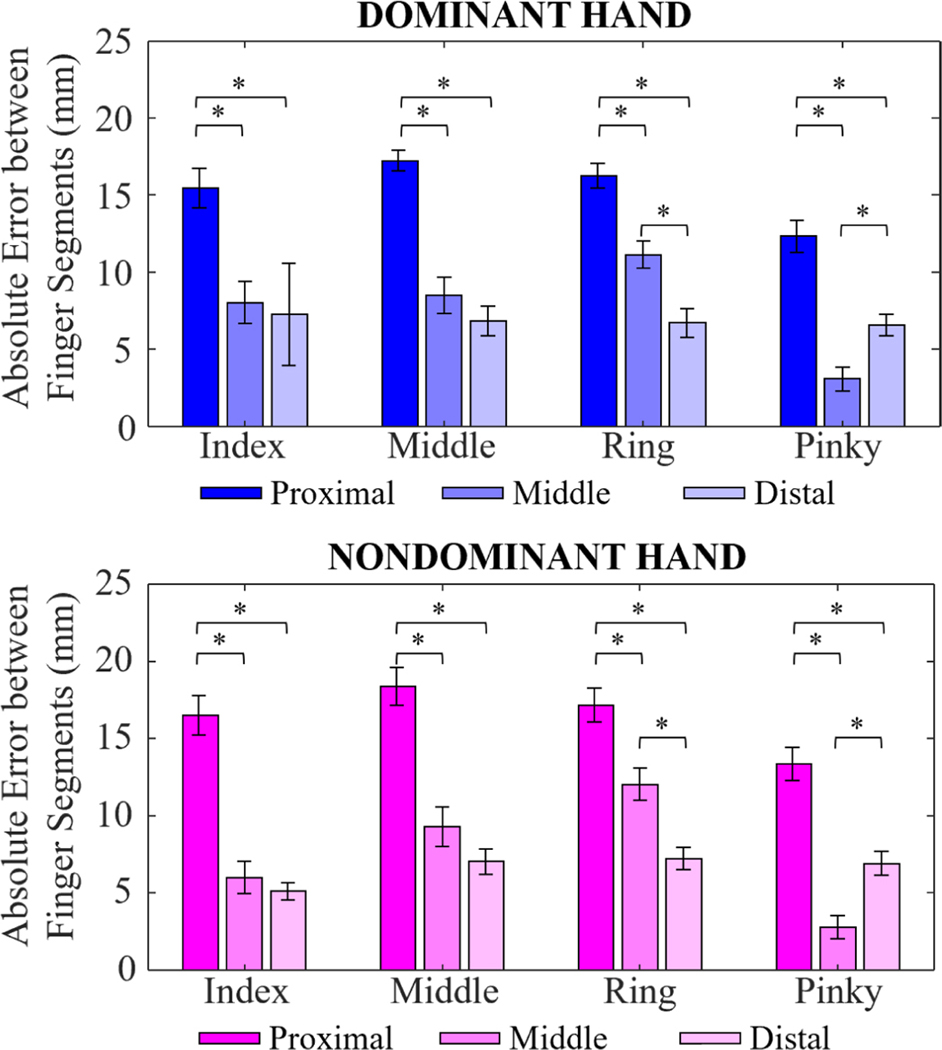
Mean absolute errors between finger segments calculated from the Leap and Optitrack hand models for the dominant (top) and nondominant (bottom) hands. * indicates statistical significance with p < 0.01, Dunn’s test for multiple comparisons.

The difference between finger segment lengths calculated from Leap and Optitrack models demonstrated that the proximal segments had larger errors than distal segments, with average errors ranging between 12.5–18.5mm (Fig. 4). Differences in segment lengths were significantly lower for the middle and distal segments, with errors as small as 2.5mm for the pinky finger of both dominant and nondominant hands.

### B. Dimensionality Reduction With PCA Demonstrates Correlation Between Low-Dimensional and High-Dimensional Spaces

**Fig. 5.**
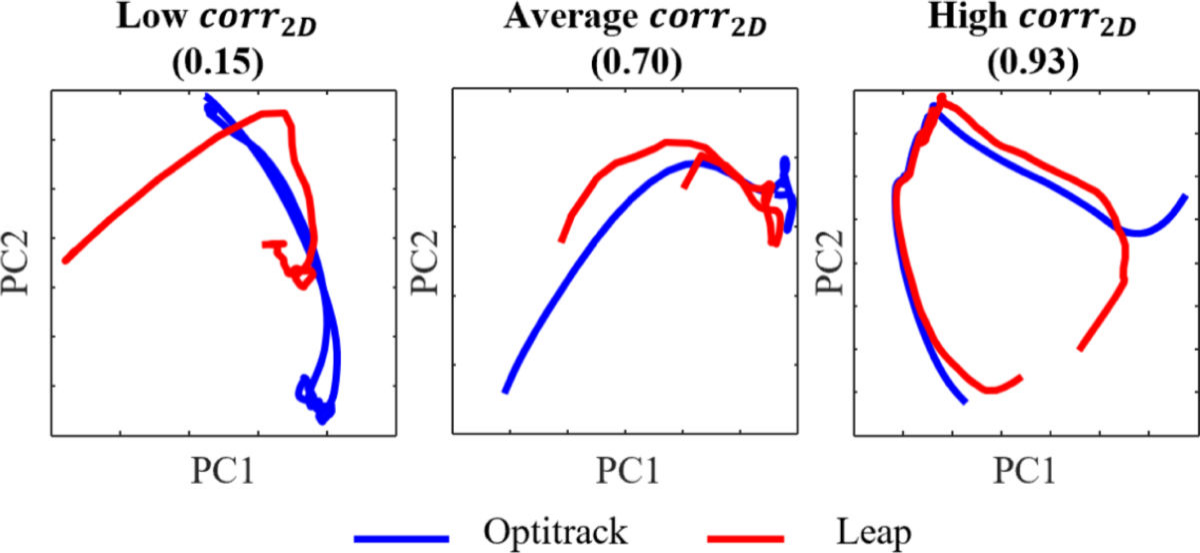
Examples of Optitrack (blue) and Leap (red) datasets encoded in a 2D latent space using PCA for the Optitrack and Leap data recorded during a single repetition of the Kapandji task by one of the participants.

Using PCA to visualize the similarity between Optitrack and Leap trajectories in a 2D space, we found that the first two components from PCA explained a high variance in the 60D for both systems, with an average variance accounted for (VAF) of 71–98% from Optitrack and Leap during the Kapandji task, and 68–98% for the finger abduction/adduction task. The correlation between the 2D latent spaces from PCA, $corr_{2D}$, was generally high with an average of 0.89 and range of 0.19–0.98 between Leap and Optitrack for both tasks. When $corr_{2D}$ values were high, latent spaces between Leap and Optitrack were more similar in shape (Fig 5). Lower $corr_{2D}$ values resulted in two encoded shapes that were very different from each other and often offset.

**Fig. 6.**
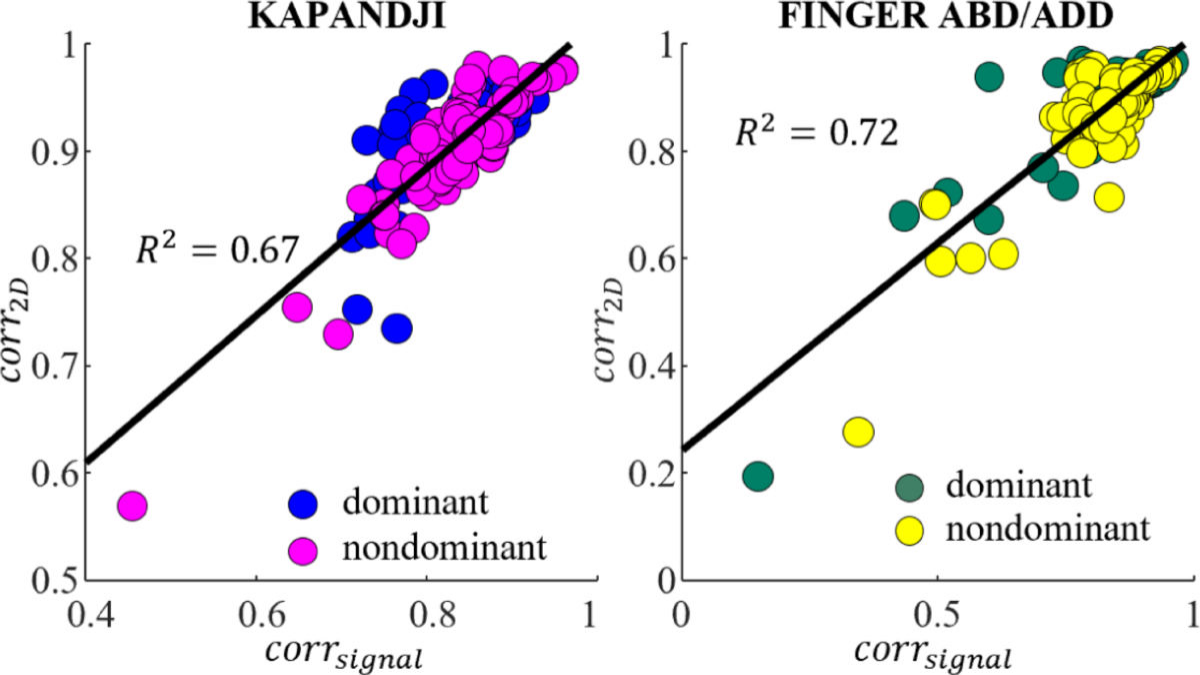
Correlation between Leap and Optitrack across all signals in the high-dimensional space $\left(corr_{signal}\right)$ versus the 2D latent spaces $\left(corr_{2D}\right)$ during Kapandji (top) and finger abduction/adduction (bottom) tasks.

To better understand how differences between the 2D latent spaces of Leap and Optitrack signified differences in the high-dimensional projections, $corr_{2D}$ values were plotted against $corr_{signal}$ (Fig. 6). Fitting a linear model yielded $R^{2}=0.67$ for Kapandji and $R^{2}=0.72$ for finger abduction/adduction, highlighting the high dependence of the shape similarity of the latent projections to the Leap-Optitrack correlation in 60D.

Additionally, when we removed the trials that had smaller VAF values, we observed an increase in the $R^{2}$ value of the linear regression model between $corr_{signal}$ and $corr_{2D}$. For example, while the baseline $R^{2}$ values were 0.67 and 0.72 for Kapandji and finger abduction/adduction, removing trials with less than 90% of VAF resulted in $R^{2}$ of 0.84 and 0.83, respectively.

## IV. Discussion

In the study, we performed a detailed analysis of Leap’s hand-tracking feature for participants with and without upper-body disabilities during tasks that were reflective of those commonly performed in clinical rehabilitation. Our results indicated high correlations that were similar regardless of the participant’s disability status. We evaluated the high-dimensional space of hand landmarks and assessed Leap’s accuracy in tracking hands, using correlation and mean absolute errors. We also evaluated the device’s ability to track the overall hand poses during the Kapandji and finger abduction/adduction tasks by evaluating the fingertip distances, reducing the original 60 dimensions to 4 functionally relevant signals for analysis. To more holistically evaluate the 60 dimensions, we also evaluated PCA for dimensionality reduction and attempted to understand how the differences in the latent projections could be impacted by original data.

### A. Accuracy of Leap’s Hand Tracking

The conventional analysis of correlation and MAE between Leap and Optitrack hand joint locations suggested that Leap’s hand-tracking features were generally acceptable during these simple dynamic tasks, although with high variability, which points to the need for new methods to monitor and evaluate signal fidelity in real-time. Mean $corr_{signal}$ values were between 0.71 and 0.94 while mean MAE was in the 10–80mm range. In the latent space identified by PCA, $corr_{2D}$ were also high (0.83–0.97) for both tasks, similarly to $corr_{signal}$ results. Most importantly, no significant differences in performance were observed between nondisabled and disabled participants. One interesting deficit in system performance was Leap’s consistent underestimation of the proximal phalanges (Fig. 4).

Our results demonstrate similar levels of accuracy for participants with disabilities as prior studies that have focused largely on nondisabled populations. The most recent study evaluated Leap’s performance with a marker-based motion capture system, Qualisys, with nondisabled participants performing thumb abduction/adduction and finger flexion/extension [20]. The reported maximum difference in the proximal segment of the index finger during static trials was lower (5.7) than average differences in segment lengths we found in our work (18mm). The trend of the proximal finger segment yielding greatest errors was similar to what we found in our study. Another study by Mizera et al. also compared Leap’s accuracy in tracking both joint angles and fingertip positions to Qualisys during random movement of the fingers and the Kapandji test in nondisabled individuals [22]. Their reported differences between computed finger segments were closer to the ranges in our study. However, the relationship of proximal segment yielding the largest errors was not observed in the study, with highest errors appearing in the distal finger segment. The study has also reported thumb-to-fingertip distance error during the Kapandji task – 8mm in comparison to the 22mm error observed in our study. However, it is important to note that distance errors were calculated during the thumb-to-fingertip contact and not over the entirety of the task as it was done in our work. The same group also evaluated Leap’s accuracy for fingertip positions and found an average positioning error of 27.4 mm during Kapandji [22], which is lower than the average MAE we calculated for all joint positions during the same task (44.4mm). These differences likely stem from the way the position errors were calculated: Mizera’s group calculated error considering only fingertip positions,we examined all joint positions and highlighted reduced accuracy for proximal segments. Other papers that assessed the accuracy of Leap have performed analysis mainly in the joint angle space [20], [21], [25] or did not report individual finger tracking [23], [24].

### B. Towards Dimensionality Reduction Analysis for Biosignal Data

Our study’s biggest motivation was to examine the applicability of dimensionality-reduction techniques for comparing complex high-dimensional biosignal data for the hand. Conventional methods fail to capture temporal delays, amplitude offsets in coordinate frames, or noise – all of which can occur during data collection in clinical environments. In conventional analyses, each signal is evaluated separately, which can be tedious to perform quickly or holistically for assessment or monitoring. In addition, different measurements are appropriate for different task conditions: while MAE is ideal for static data analysis (*i.e*., single time instance), correlation is great for dynamic tasks (*i.e*., data over time).

Dimensionality-reduction algorithms can prove to be useful by efficiently compressing high-dimensional data into more manageable, lower-dimensional spaces. Such methods can improve the interpretability of results. In our study, we saw that compressing 60D joint position data to 2D via PCA produced meaningful results, where large differences in the original high-dimensional space were reflected in the differences between Leap and Optitrack projections in 2D (Fig. 6). The results produced by evaluating $corr_{2D}$, the correlations of latent projections, yielded results similar to those obtained when evaluating all the signals in high dimensions, $corr_{signal}$. There, the $corr_{signal}$ range was 0.71–0.94 while mean while $corr_{2D}$ range was (0.83–0.97). Small discrepancies in the results could be attributed to PCA’s inability to effectively compress all the important information in every trial. As noted, average VAF was as low as 71%. Lastly, in our exploration, we found that PCA can account for similar data injected with noise, amplitude offset, and temporal delay, making it a useful tool in performing comparison of high-dimensional biosignal data.

Dimensionality reduction has a rich history in evaluating the hand and other physiological signals [33], [34], [35], [36], [44], [45], [46], [47]. In the past, it has been utilized to capture hand kinematics during object grasping and gesturing, leg kinematics of human gait, as well as muscle activations during various tasks, with the main objective of reducing the complexity of high-dimensional biological signals. Oftentimes, these applications focused on the derivation of “synergies”, or control units. In this study, however, we leveraged the DR method for a novel application of comparing high-dimensional physiological signals in a lower-dimensional space, with the potential to monitor hand function.

As with any tool, there are limitations to the use of DR techniques for the purpose of accuracy assessment. First, for certain trials, VAF by first two components was moderate, with values as low as 68%. Generally, this level of variance is considered acceptable, although it is important to understand that the other 32% of variance are not captured in the latent space. Such performance is tied to PCA’s objective to force orthogonality of latent dimensions (principal components) and the assumption of linearity. Given the anatomy of the hand and the tasks performed, there are expected non-orthogonal and dependent components in these actions. Other DR techniques that do not require these constraints, such as nonnegative matrix factorization, may be more appropriate, although they often lack the interpretability of PCA. When trials with lower VAF scores were removed from latent space analysis, we found a stronger linear relationship between the correlation of high-dimensional signals and the correlation of latent representations in 2D. Noting the challenges of compression that PCA sometimes exhibits with complex data, it is essential to remember that it is only one of many DR techniques, some of which (*e.g*., nonlinear autoencoders) have been shown to be significantly more efficient at compressing complex hand kinematics data than their linear counterparts [36], [47]. In such cases, VAF values tend to be in the 90% range. Analysis of the latent spaces produced with such techniques could potentially be more reliable and indicative of the original high-dimensional space. While our initial exploration with PCA is promising, future work should consider more complex DR techniques. Additionally, PCA’s main objective does not lie in preserving relative distance between data points but with the overall variance along axes. However, preserving local geometries in the case of signal accuracy assessment might be an important aspect and other DR techniques whose goal is to preserve local structures should be considered and evaluated in the future (e.g., Isomap [44], Locally Linear Embedding [48]). Lastly, PCA does not account for the temporal aspect of the original data, so that each data point on the latent space signifies a single sample in the high-dimensional space. In the future, temporal DR techniques, such as the ones that rely on long short-term memory [49], could be explored to evaluate data from dynamic tasks in a more efficient way and complex time-dependent data could be reduced to a single point.

### C. Study Limitations

In this study, we wanted to capture a variety of disabilities that affected hand function. As a result, we had broad inclusion criteria, the participant pool was not homogeneous, and certain disability types appeared more often than others. Another limitation comes from the space allocated for the experiment – motion capture was performed in a small room, making it difficult to place cameras to prevent occlusion. Mobility aids also resulted in greater marker occlusions, often losing markers for a few frames. In such cases, the lost marker position was estimated from other markers using regression or pattern recognition techniques. We operated the Leap device in a desktop recording mode, where it is placed flat on the table and the hands are only recognized when in the pronate position (palm down towards the device). On the contrary, for Optitrack, markers were placed on top of the hand, since placing motion capture cameras under the table was impossible due to space limitations and accommodating wheelchairs. As a result, Optitrack joint position tracking happened from the top of the hand while Leap performed image recognition from the bottom of the hand. Gemini, the hand-tracking software, can also operate in “head-mounted” and “screentop” modes, which may impact accuracy and warrant further investigation for hand tracking and assessment. Lastly, the hand tasks selected for data analysis in our study, such as the modified Kapandji and finger abduction/adduction, while are representative of common device interactions and similar to hand assessments for range of motion, cannot be generalized to tasks that involve object manipulation used in hand rehabilitation.

### D. Looking Ahead

Our study has shown that existing off-the-shelf camera-based solutions for hand tracking have a promising potential for novel ways to perform hand rehabilitation. Leap was determined to be an inclusive technology for individuals with different upper-body disabilities. In addition, our work with DR techniques to compare high-dimensional data for accuracy assessment can potentially be used to provide real-time monitoring or biofeedback during and after therapy. In the past, availability of biosignals during rehabilitation exercises has been shown to improve outcomes [50], [51], [52].

Analyses using the latent space analysis may also have the potential to provide insight into issues or changes – such as to track progress across sessions or monitor for disease progression. Similarly, these representations can prove to be useful not only in the upper-limb but in other physiological domains. For example, in the past, PCA has been used to evaluate and detect abnormalities in breathing [53] and human gait [54], [55] and help identify emergency situations in patients from mobile health application data [56], [57]. The overall good and reliable performance of the Leap relative to optical motion capture in participants with and without disabilities in this study suggests that these hand-tracking technologies provide promising and inclusive strategies to deploy in rehabilitation and assistive technology.

## V. Conclusion

This paper presents an accuracy assessment of the Leap’s hand tracking feature for individuals with upper-body disabilities. The feature performed similarly regardless of the disability status of the user, with variable results for participants with and without disabilities. In addition, the paper proposes the use of a linear dimensionality-reduction technique, PCA, to be used as a tool to compare high-dimensional data on a latent space. Following the analysis, we determined that high correlations between lower-dimensional projections were associated with high correlations of the data in the original high-dimensional space, suggesting the potential of PCA to be used for comparative purposes of high-dimensional data.

## Supplementary Material

Supplemental1-3398610
