# Supplementary material for: Accuracy of Video-Based Hand Tracking for People With Upper-Body Disabilities
Source: IEEE Trans Neural Syst Rehabil Eng. Author manuscript; Available in PMC 2025 May 24. (PMC12103098; doi:10.1109/TNSRE.2024.3398610)
Supplement: Supplemental1-3398610 [file NIHMS2069451-supplement-Supplemental1-3398610.docx]

^^[[1]](#footnote-1)^^

Supplementary Material


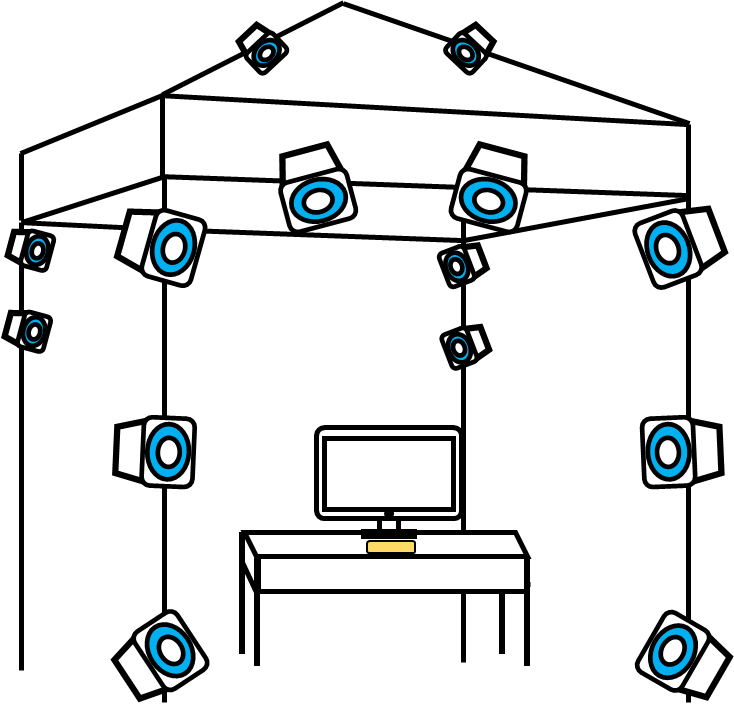


Supplementary Fig. 1. Experimental setup with 14 Prime^x^ 13W cameras placed on a 6x6ft canopy. The cameras faced a desk at which the participants were seated in front of a computer display. The Leap device was placed along the edge of the table closest to the participants.


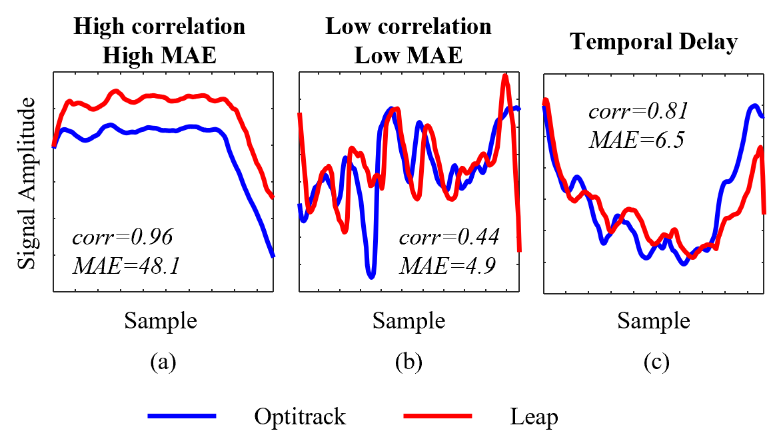


Supplementary Fig. 2. Examples of two signals with (a) high correlation/high MAE, (b) low correlation/low MAE, and (c) temporal delay, selected from representative data from P111 and P119 during the Kapandji task.


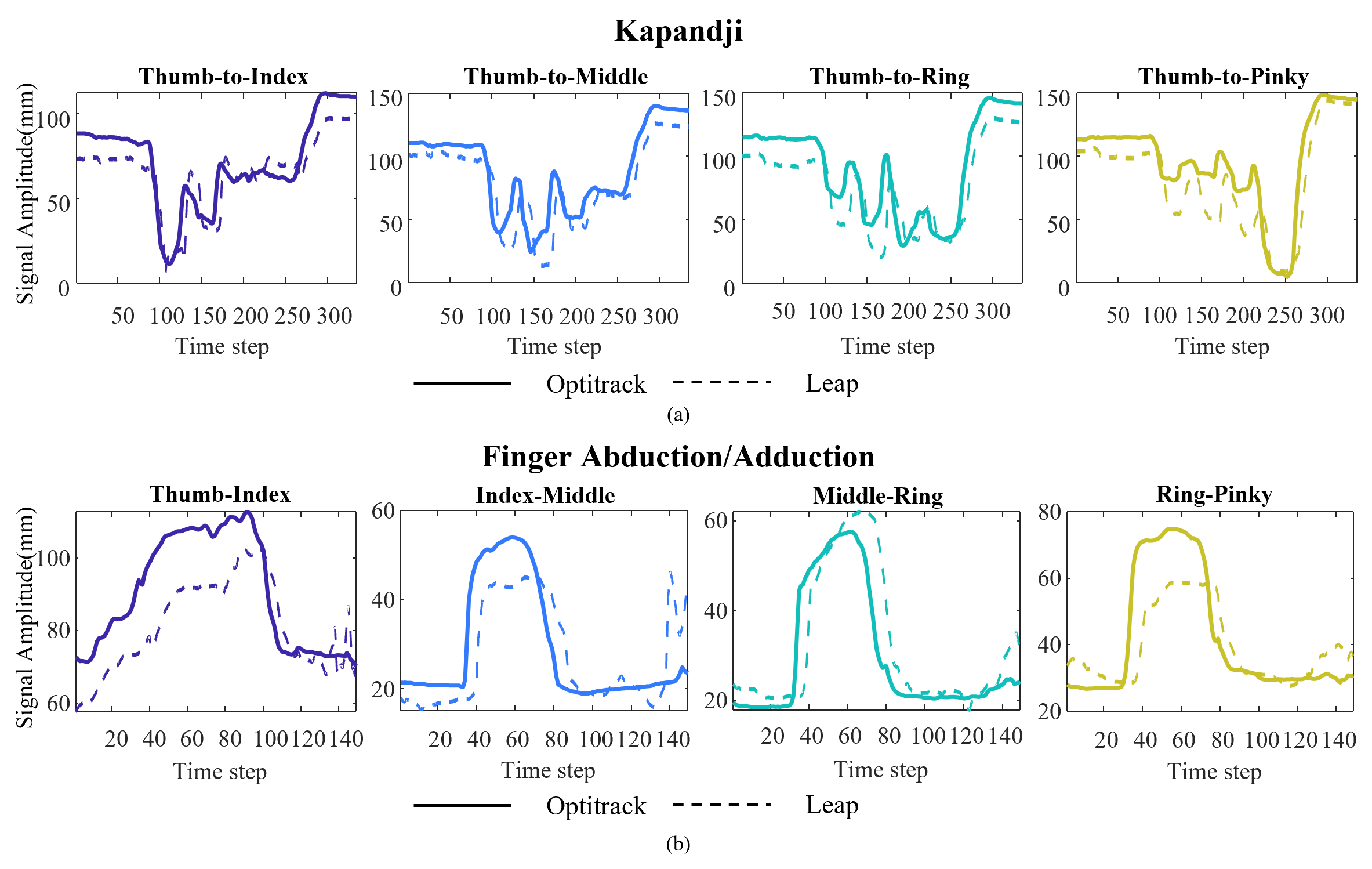


Supplementary Fig. 3. The Euclidian distances between digit tips were evaluated to reduce dimensionality of the degrees of freedom for analysis and capture functional elements often used for assessment and monitoring of hand function. Representative data from are shown for (a) the thumb and each finger during Kapandji (P105) and (b) between adjacent digits during Finger Abduction/Adduction (P116).


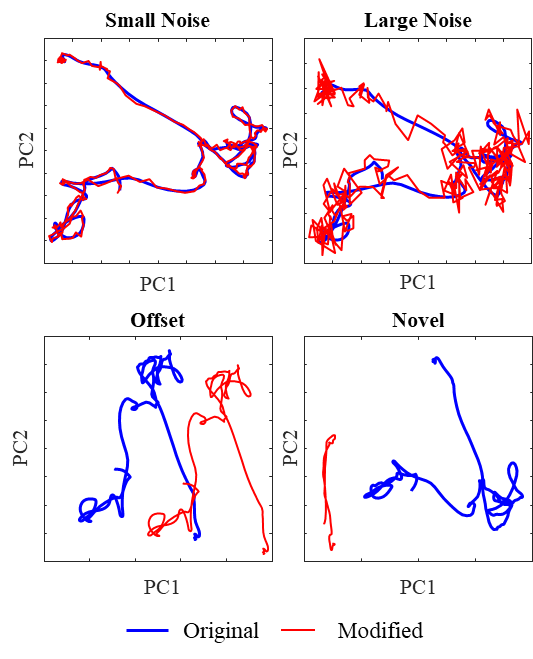


Supplementary Fig. 4. Latent spaces of two datasets combined into one, with one of the datasets being original while the other was either a modification of the original one (with small noise, large noise, amplitude offset) or a completely novel data.

1. [↑](#footnote-ref-1)
